# Mammalian, plant, and gut microbiota-derived extracellular vesicles as emerging therapeutics for bone diseases

**DOI:** 10.3389/fbioe.2026.1825617

**Published:** 2026-05-08

**Authors:** Guanchen Yin, Hao Wang, Yi Liu, Zhenyan Wang, Xu Lin, Qisheng Cheng, Hang Gao

**Affiliations:** 1 Department of Bone and Joint Surgery, Orthopaedic Surgery Center, The First Hospital of Jilin University, Changchun, China; 2 Department of Orthopedics, Yantai Yantaishan Hospital, Yantai, China

**Keywords:** bone, extracellular vesicles, gut microbiota-derived extracellular vesicles, mammalian cell-derived extracellular vesicles, plant-derived extracellular vesicles

## Abstract

Bone disorders, including osteoporosis and osteoarthritis, represent a growing global health burden, yet current therapies remain limited by poor targeting efficiency and significant adverse effects. Extracellular vesicles (EVs), nanoscale lipid bilayer particles that mediate intercellular communication, have emerged as promising endogenous nanocarriers with intrinsic biocompatibility and regulatory capacity. This review summarises recent advances in EVs-based therapeutic strategies for bone diseases, focusing on EVs derived from mammalian cells, plants, and the gut microbiota. Particular emphasis is placed on gut microbiota-derived EVs, which have gained increasing attention for their role in regulating bone homeostasis through the “gut-bone axis”. By comparing the biological characteristics, advantages, limitations, and disease-specific applicability of EVs from these distinct sources, we highlight their complementary therapeutic potential. Overall, this review provides a concise framework for the rational development and clinical translation of EVs-based precision therapies for bone disorders.

## 1 Introduction

Bone disorders—including osteoporosis (OP), osteoarthritis (OA), rheumatoid arthritis (RA), fractures, and avascular necrosis of the femoral head—are increasingly prevalent worldwide and constitute a major cause of disability. Global burden of disease analyses identify musculoskeletal disorders as one of the leading contributors to years lived with disability, reflecting the substantial clinical and socioeconomic impact of these conditions. Population aging and lifestyle changes further exacerbate this burden, underscoring the need for more effective therapeutic strategies (GBD, 2021 Diseases and Injuries Collaborators, 2024; GBD, 2021 Other Musculoskeletal Disorders Collaborators, 2023).

Current treatments rely primarily on pharmacological intervention and surgery, yet their long-term efficacy is limited. In OP, anti-resorptive and anabolic agents act unidirectionally on bone remodeling and often fail to restore physiological balance, while prolonged use is associated with poor adherence and serious adverse effects (Ayers et al., 2023; Bellido, 2024; Reid and Billington, 2022). OA management remains largely symptomatic, with non-steroidal anti-inflammatory drugs and joint replacement surgery constrained by systemic toxicity, surgical risk, and limited disease-modifying capacity (Zeng et al., 2018; Richardson et al., 2023). Similar challenges are evident across other bone pathologies, highlighting the unmet need for targeted, mechanism-based therapies (Serhal et al., 2020; Depypere et al., 2020). In addition to traditional drug and surgical treatments, therapies related to the “gut-bone axis” are also gaining increasing attention. The traditional “gut-bone axis” primarily involves the direct regulation of bone metabolism by gut microbiota metabolites (e.g., SCFAs), as well as indirect regulation via the host’s immune and endocrine systems. Based on these mechanisms, traditional “gut-bone axis” therapies, such as dietary interventions, probiotics, and fecal microbiota transplantation (FMT), have been applied to the treatment of bone diseases (Liu et al., 2025). However, despite their therapeutic potential, these interventions still face significant clinical challenges, including the risk of colonization failure and systemic infection (Yadegar et al., 2024).

In recent years, addressing the clinical challenges of bone regeneration has driven the extensive exploration of advanced biomaterials and therapeutic strategies to enhance bone regeneration and implant osseointegration. Significant progress has been made in engineering functional microenvironments for bone repair, including the development of biomimetic periosteum (Du et al., 2025), tissue-adhesive barrier membranes for guided bone regeneration (GBR) (Poos et al., 2025), and the application of nanotechnology to optimize orthopedic implants and composite scaffolds (Liang et al., 2024). In this context, extracellular vesicles (EVs) have emerged as a highly promising new alternative and adjunctive therapeutic approach in the field of orthopedics (Kumar et al., 2024; Liu Z. et al., 2023; Fang et al., 2024; Liu H. et al., 2022). Nearly all eukaryotic and prokaryotic cells secrete EVs, which are nanoscale particles (typically 30–1,000 nm in diameter) enclosed by a robust lipid bilayer and serve as fundamental mediators of intercellular and interorgan communication. This stable membrane structure effectively protects the complex bioactive substances within (including nucleic acids, proteins, lipids, and metabolites) from enzymatic degradation, facilitating their functional delivery to recipient cells via endocytosis, direct membrane fusion, or receptor-ligand interactions. Compared to synthetic nanocarriers, naturally derived EVs exhibit excellent biocompatibility, extremely low immunogenicity, and an inherent ability to evade immune clearance and traverse biological barriers. Consequently, they serve as highly effective and safe delivery vehicles for the treatment of systemic and localized skeletal disorders (Welsh et al., 2024; Huang et al., 2023; van Niel et al., 2022; Yin et al., 2022).

Growing evidence implicates EVs in the regulation of bone metabolism, inflammation, and regeneration (Liu et al., 2021; Thomas et al., 2024; Chen et al., 2022). While mammalian cell-derived and plant-derived EVs have been extensively studied, EVs derived from the gut microbiota have only recently attracted attention (Fan and Pedersen, 2021). The gut microbiota plays a central role in skeletal homeostasis through the “gut-bone axis”, integrating immune, metabolic, and endocrine signaling (Welsh et al., 2024; Huang et al., 2023). Notably, gut microbiota-derived EVs(GM-EVs) can cross the intestinal barrier, enter systemic circulation, and influence distant tissues, including bone, thereby representing a critical mediator of microbiota-host crosstalk (Chen et al., 2022; Jiao et al., 2025).

In this review, we provide a concise and comparative overview of EVs derived from mammalian cells, plants, and the gut microbiota in bone diseases. We highlight the emerging role of GM-EVs, contrast the strengths and limitations of EVs from different origins, and discuss their translational potential. This synthesis aims to inform the rational design of EVs-based therapeutic strategies for bone disorders.

## 2 Mammalian cell-derived extracellular vesicles and their role in bone diseases

Mammalian cell-derived EVs (MEVs), released by mesenchymal stem cells (MSCs), immune cells, endothelial cells, and bone-resident cells, are key regulators of skeletal homeostasis (Giancaterino and Boi, 2023). These EVs regulate osteoblast (OB) differentiation, osteoclast (OC) activity, and osteocyte signaling, thereby coordinating the processes of bone formation and resorption (Ra et al., 2010; Huang Y. et al., 2021; Lu et al., 2019). For example, studies demonstrate that EVs derived from bone marrow mesenchymal stem cells (BMSCs) can deliver lncTUG1 to OB, sequestering miR-22-5p and upregulating Anxa8 expression, thereby promoting OB differentiation and accelerating fracture healing (Li et al., 2023). Furthermore, BMSCs-derived EVs containing miR-206 enhance OB proliferation and differentiation in OA by downregulating Elf3, contributing to the attenuation of OA progression (Huang Y. et al., 2021). Similarly, EVs derived from adipose-derived MSCs (ADSCs) alleviate diabetic OP by inhibiting NLRP3 inflammasome activation in OC (Zhang L. et al., 2021). In addition, Davies et al. found that EVs released by mineralized OB promote MSCs differentiation into OB by carrying Annexin A1, A2, and A6, and bridging collagen type VI (Davies et al., 2017).

**FIGURE 1:**
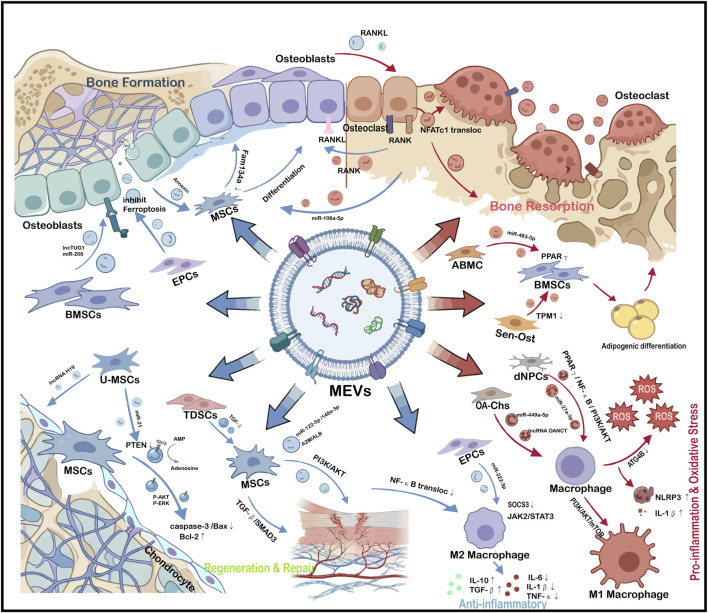
MEVs in the Regulation of Bone Homeostasis. Taken together, this figure illustrates the crucial bidirectional regulatory role of MEVs in skeletal homeostasis: they actively drive OB-mediated bone formation and tissue/angiogenic repair, while simultaneously suppressing osteoclastogenesis, adipogenic differentiation, and macrophage-mediated inflammatory and oxidative stress responses.

MEVs exert bidirectional effects on bone metabolism. EVs derived from young or healthy cells generally promote osteogenesis, suppress osteoclastogenesis, and facilitate fracture repair, whereas EVs originating from aged, senescent, or diseased cells may impair osteogenic differentiation, enhance adipogenesis, and exacerbate bone loss (Wang et al., 2024; Ni et al., 2019; Lv et al., 2022; Zhao et al., 2023). Evidence indicates that EVs isolated from the plasma of OP patients suppress MSCs differentiation into OB via miR-1246. Furthermore, they promote OC activation by presenting surface RANKL and upregulating RANKL expression in OB, shifting the remodeling equilibrium toward excessive resorption (Pepe et al., 2022). Beyond metabolic regulation, MEVs modulate inflammatory and oxidative stress responses by shaping macrophage polarization, regulating T-cell subsets, and controlling key pathways such as NF-κB, PI3K-AKT, STAT3, and Nrf2 (Li et al., 2022; Yuan et al., 2023; Chen et al., 2024; Li Y. et al., 2024). For instance, EVs derived from human umbilical cord MSCs (hUCMSCs) deliver miR-122-5p, miR-148a-3p and key proteins including A2M and ALB to activate the PI3K-Akt pathway. This activation suppresses NF-κB nuclear translocation, promotes M2 macrophage polarization, reduces pro-inflammatory cytokines including IL-6, IL-1β and TNF-α, and increases anti-inflammatory cytokines including IL-10 and TGF-β, thereby alleviating OA (40). Similarly, EVs derived from H_2_O_2_-pretreated ADSCs significantly reduce ROS levels and oxidative damage in diabetic BMSCs by activating the Nrf2/HO-1 pathway. This alleviates cellular senescence and enhances osteogenic differentiation, promoting the repair of diabetic bone defects (Li Y. et al., 2024). In addition, MEVs support tissue regeneration by promoting angiogenesis, inhibiting apoptosis, remodeling extracellular matrix, and activating resident progenitor cells (Chew et al., 2019; Yan et al., 2021; Kuang et al., 2019; Liu et al., 2017; Xu et al., 2019; Wu et al., 2023). Specifically, umbilical cord MSC-derived EVs deliver miR-21 to target PTEN mRNA, suppressing PTEN protein expression. This alleviates the inhibition of the AKT pathway and promotes AKT phosphorylation, leading to reduced expression of apoptotic proteins such as cleaved Caspase-3 and Bax and increased expression of the anti-apoptotic protein Bcl-2. Consequently, these EVs attenuate osteocyte apoptosis and ameliorate the severity of osteonecrosis of the femoral head in rat models (Kuang et al., 2019). In addition,MSC-derived EVs enhance endothelial cell proliferation, migration, and tube formation capacity by activating the PI3K/AKT pathway, thus promoting angiogenesis and offering a promising strategy for the prevention and treatment of osteonecrosis of the femoral head (Liu et al., 2017). Owing to their high biocompatibility, intrinsic targeting capacity, and cargo versatility, MEVs represent a promising platform for precision therapies in chronic bone diseases (as summarized in Figure 1; Table 1). However, translating positive preclinical findings into clinical practice requires careful consideration. A major limitation is that current mechanistic studies rely heavily on small animal models, which cannot fully replicate the complex long-term dynamics of skeletal remodeling in humans. Furthermore, the inherent batch-to-batch variability in cell culture and the lack of standardized protocols for isolating clinical-grade MEVs remain key bottlenecks that must be overcome before large-scale clinical translation can be achieved.

**TABLE 1:** Summary of key cargo molecules in MEVs and their functions and regulatory mechanisms in skeletal diseases.

EVs type	EVs source	Molecular mediators in the EVs	Function and mechanism of action	References
MEVs	BMSCs	LncTUG1	Competitively binds miR-22-5p to upregulate Anxa8, promoting OBs differentiation and fracture healing	Li et al. (2023)
	BMSCs	miR-206	Downregulates Elf3 to enhance OBs proliferation/differentiation and delay OA progression	Huang Y et al. (2021)
	U-MSCs	LncRNA H19	Promotes chondrocyte proliferation and matrix synthesis while inhibiting apoptosis	Chen et al. (2024)
	U-MSCs	miR-21	Inhibits PTEN to activate AKT, decreasing Bax/Caspase-3 and increasing Bcl-2 to reduce osteocyte apoptosis	Li Y et al. (2024)
	U-MSCs	miR-122-5p, miR-148a-3p, A2M, ALB	Activates the PI3K-Akt pathway, inhibits NF-κB, and promotes M2 macrophage polarization, thereby alleviating OA	Lv et al. (2022)
	TDSCs	TGF-β	Activates TGF-β/SMAD3 to promote tenogenic differentiation and stem cell proliferation for tendon regeneration	Yan et al. (2021)
	EPCs	miR-222-3p	Downregulates SOCS3 to activate JAK2/STAT3, promoting M2 macrophage polarization and alleviating spinal cord inflammation	Zhao et al. (2023)
	EPCs	-	Inhibiting OBs ferroptosis to improve hormone-induced OP and osteonecrosis	Lu et al. (2019)
	OBs	Annexin A1/A2/A6	Binds to type VI collagen, thereby promoting the differentiation of MSCs into OBs	Davies et al. (2017)
MEVs	OCs	miR-106a-5p	Downregulates Fam134a, enhances osteogenic differentiation of BMSCs, and thereby promotes bone regeneration	Kuang et al. (2019)
	ABMC	miR-483-5p	Targets PPARγ to promote BMSCs adipogenesis, inducing bone-fat imbalance and OP	Wang et al. (2024)
	OA-Chs	miR-449a-5p	Inhibits ATG4B to block autophagy, inducing mitochondrial ROS and NLRP3 inflammasome activation to exacerbate OA.	Ni et al. (2019)
	OA-Chs	LncRNA OANCT	Inhibits m^6^A demethylation to stabilize PIK3R5 mRNA, activating PI3K/AKT/mTOR and M1 macrophage polarization to exacerbate OA	Lv et al. (2022)
	dNPCs	miR-27a-3p	Activates the PPARγ/NF-κB/PI3K/AKT pathway, induces M1 macrophage polarization, thereby exacerbating intervertebral disc degeneration	Zhao et al. (2023)
	Sen-Ost	-	Reducing TPM1 expression promotes the adipogenic differentiation of BMSCs	Wang et al. (2024)

BMSCs, Bone Marrow Mesenchymal Stem Cells; U-MSCs, Mesenchymal Stem Cells; TDSCs, Tendon-Derived Stem Cells; EPCs, Endothelial Progenitor Cells; OBs, Osteoblasts; OCs, Osteoclasts; ABMC, Aged Bone Matrix Cells; OA-Chs, Osteoarthritis Chondrocytes; dNPCs, Degenerated Nucleus Pulposus Cells; Sen-Ost, Senescent Osteocytes; LncTUG1, Long Non-Coding RNA TUG1; Anxa8, Annexin A8; Elf3, E74-Like Factor 3; OA, Osteoarthritis; LncRNA H19, Long Non-Coding RNA H19; PTEN, Phosphatase and Tensin Homolog; AKT, Protein Kinase B; Caspase-3, Cysteine Aspartate Protease-3; Bax, Bcl-2-Associated X Protein; Bcl-2, B-Cell Lymphoma 2; A2M, Alpha-2-Macroglobulin; ALB, Albumin; SOCS3, Suppressor of Cytokine Signaling 3; PPARγ, Peroxisome Proliferator-Activated Receptor Gamma; ATG4B, Autophagy-Related Gene 4B; ROS, Reactive Oxygen Species; m^6^A, N6-Methyladenosine; TPM1, Tropomyosin-1.

## 3 Plant EVs and bone disorders

Plant-derived EVs (PDEVs), naturally secreted by edible and medicinal plants, encapsulate a rich repertoire of plant-specific phytochemicals, lipids, proteins, and regulatory RNAs. Endowed with intrinsic anti-inflammatory, antioxidant, and regenerative properties, PDEVs are emerging as a highly compelling natural nanotherapeutic strategy for the management of bone disorders (Cui et al., 2020; Lian et al., 2022).

In bone diseases, PDEVs promote osteogenic differentiation, inhibit OC activation, and rebalance bone remodeling by activating signaling pathways such as BMP-Runx2, MAPK, and Smad (Zhan W et al., 2023; Hwang et al., 2023; Cao et al., 2024; Park et al., 2023; Seo et al., 2023). Specifically, yam-derived EVs (YNVs) activate the BMP-2/p-p38-dependent Runx2 signaling pathway, thereby promoting OB proliferation, differentiation, and mineralization, ultimately improving bone mineral density and microarchitectural integrity in osteoporotic mice (Hwang et al., 2023). Furthermore, euphorbia-originated EVs (MOEVLPs) promote the proliferation of osteoblastic precursor cells (MC3T3-E1) by activating the MAPK signaling pathway, leading to enhanced bone formation and improved trabecular structure in OP models (Cao et al., 2024). Concurrently, PDEVs attenuate inflammation and oxidative stress by suppressing NF-κB and NLRP3 inflammasome activation, activating Nrf2-dependent antioxidant responses, and modulating macrophage polarization (Kim et al., 2023; De Robertis et al., 2020; Liu C. et al., 2022; Zeng et al., 2024; Lou et al., 2023). In particular, ginger-derived EVs relieve Keap1-mediated inhibition of Nrf2 and promote Nrf2 nuclear translocation, thereby downregulating inflammatory factors including IL-6 and TNF-α and upregulating antioxidant genes such as heme oxygenase-1 (HO-1) and NAD(P)H quinone dehydrogenase 1 (NQO1) to reduce ROS and oxidative damage (Zeng et al., 2024). In tandem, grapefruit-derived EVs improve the chondrocyte microenvironment by downregulating inflammatory genes including COX2 and PTGS2 and upregulating antioxidant genes including superoxide dismutase 2 (SOD2) and glutathione peroxidase (GPX), exerting combined anti-inflammatory and antioxidant effects (Rashidi et al., 2025).

**FIGURE 2:**
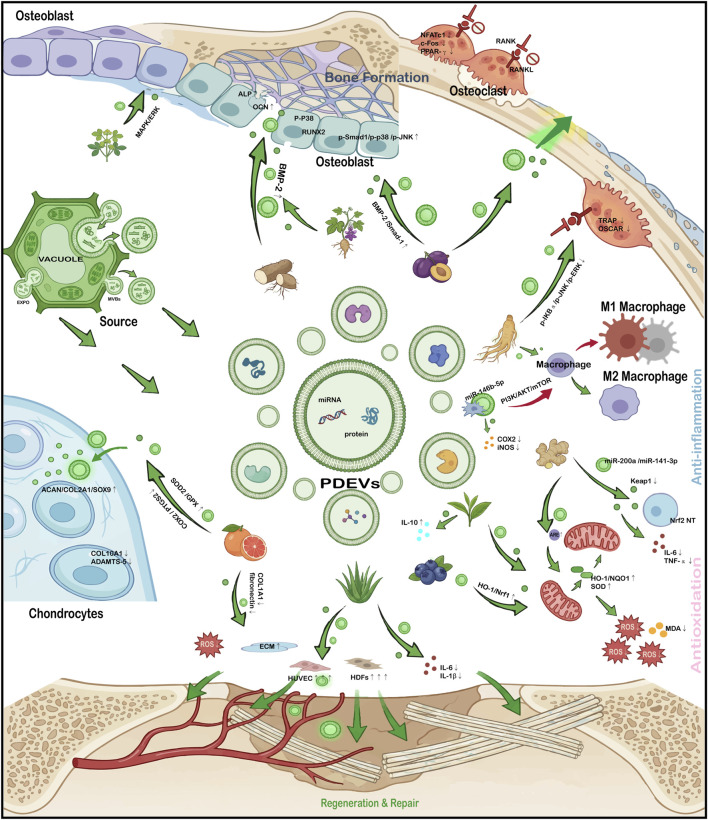
PDEVs in the Regulation of Bone Homeostasis. Collectively, this figure highlights the comprehensive therapeutic potential of PDEVs, demonstrating their ability to orchestrate bone and cartilage regeneration, promote angiogenesis for tissue repair, and fundamentally improve the local microenvironment through potent, multi-targeted anti-inflammatory and antioxidant signaling.

Although direct evidence for PDEVs-mediated bone regeneration remains limited, their ability to improve the inflammatory, oxidative, and angiogenic microenvironment indirectly supports skeletal repair (Rashidi et al., 2025; Zu et al., 2021). Furthermore, grapefruit-derived EVs accelerate wound healing through their antioxidant effects by reducing ROS generation. They also stimulate extracellular matrix (ECM) production by upregulating COL1A1, fibronectin, and other related genes, and promote angiogenesis by enhancing the tube-forming capacity of vascular endothelial cells (Savcı et al., 2021). Similarly, Aloe vera-derived EVs exhibit anti-inflammatory properties by suppressing mRNA expression of pro-inflammatory cytokines including IL-6 and IL-1β, promote angiogenesis by improving endothelial cell tubulogenesis, and enhance fibroblast proliferation and migration, collectively offering therapeutic value in chronic wound healing (Kim and Park, 2022). PDEVs are therefore well suited as adjunctive therapies for multifactorial bone disorders, such as OA and OP, particularly where inflammation and oxidative stress are dominant drivers of pathology (as summarized in Figure 2; Table 2). Although the multifunctionality of PDEVs has been well documented *in vitro* and in animal models, there remains a significant gap in understanding their direct clinical relevance in targeted bone therapy. Furthermore, current research primarily relies on local administration or nonspecific systemic delivery. Whether orally administered PDEVs can naturally bypass the gastrointestinal barrier and reach therapeutic concentrations in distant skeletal sites remains an unproven hypothesis. These highlight key areas for future research.

**TABLE 2:** Summary of key cargo molecules in PDEVs and their functions and regulatory mechanisms in skeletal diseases.

EVs type	EVs source	Molecular mediators in the EVs	Function and mechanism of action	References
PDEVs PDEVs	Euphorbia	-	Activates MAPK/CREB/RSK1 signaling to promote MC3T3-E1 proliferation and ameliorate OP	Cao et al. (2024)
	Yam	-	Activates BMP-2/p-p38/Runx2 pathway to enhance OBs proliferation, differentiation, and mineralization	Hwang et al. (2023)
	Plum	-	Upregulates BMP-2/Smad-1 and p-p38/p-JNK to induce Runx2/Osterix-mediated osteogenesis; downregulates NFATc1/c-Fos to block RANKL-induced osteoclastogenesis	Park et al. (2023)
	Puerariae lobata	-	Increases Runx2 levels to drive ALP/OCN expression and promote BMSC osteogenic differentiation	Zhan H et al. (2023)
	Ginseng	-	Inhibits IκBα/JNK/ERK phosphorylation to downregulate NFATc1/c-Fos and block OCs maturation	Seo et al. (2023)
	Fucosylated MSCs	miR-146b-5p	Blocks PI3K/AKT/mTOR to reduce COX-2/iNOS, inhibit M1 macrophages polarization, and alleviate OA	Lou et al. (2023)
	Ginger	-	Inhibits Keap1 to promote Nrf2 nuclear translocation, upregulating HO-1/NQO1 and reducing oxidative damage	Zeng et al. (2024)
	Tea	-	Reduces ROS and pro-inflammatory factors while promoting IL-10 secretion	Zu et al. (2021)
	Blueberry	-	Upregulates HO-1/NQO1 and reduces ROS levels	De Robertis et al. (2020)
	Grapefruit	-	Reduces ROS and upregulates COL1A1/Fibronectin to promote ECM formation and angiogenesis	Savcı et al. (2021)
	Grapefruit	-	Downregulates COX2/PTGS2 and upregulates SOD2/GPX to improve chondrocyte microenvironment	Rashidi et al. (2025)
	Aloe vera	-	Suppresses IL-6/IL-1β, promotes angiogenesis, and enhances fibroblast migration for chronic wound healing	Kim and Park (2022)

MAPK, Mitogen-Activated Protein Kinase; CREB, cAMP Response Element-Binding Protein; RSK1, Ribosomal S6 Kinase 1; MC3T3-E1, Mouse Osteoblast Cell Line; BMP-2, Bone Morphogenetic Protein 2; p38, p38 Mitogen-Activated Protein Kinase; Runx2, Runt-Related Transcription Factor 2; OBs, Osteoblasts; OCs, Osteoclasts; Smad1, Mothers Against Decapentaplegic Homolog 1; JNK, c-Jun N-Terminal Kinase; Osterix, Sp7 Transcription Factor; ALP, Alkaline Phosphatase; OPN, Osteopontin; OCs, Osteoclasts; NFATc1, Nuclear Factor of Activated T Cells 1; c-Fos, Proto-Oncogene c-Fos; PPAR-γ, Peroxisome Proliferator-Activated Receptor Gamma; OCN, Osteocalcin; BMSCs, Bone Marrow Mesenchymal Stem Cells; TRAP, Tartrate-Resistant Acid Phosphatase; OSCAR, Osteoclast-Associated Receptor; MSCs, Mesenchymal Stem Cells; COX-2, Cyclooxygenase-2; iNOS, Inducible Nitric Oxide Synthase; OA, Osteoarthritis; HO-1, Heme Oxygenase 1; NQO1, NAD(P)H Quinone Dehydrogenase 1; ROS, Reactive Oxygen Species; COL1A1, Collagen Type I Alpha 1; ECM, Extracellular Matrix; PTGS2, Prostaglandin-Endoperoxide Synthase 2; SOD2, Superoxide Dismutase 2; GPX, Glutathione Peroxidase.

## 4 Gut microbiota EVs and bone disorders

Bone disorders are often associated with intestinal dysbiosis. For instance, OP patients show elevated levels of *Clostridium*, *Ruminococcaceae*, and *Megamonas* but reduced *Roseburia* and *Weissella* (Yang et al., 2022). Similarly, OA patients exhibit increases in *Roseburia* and *Butyricicoccus* alongside decreases in *Bacteroides spp*. and *Faecalibacterium prausnitzii* (Wang W. et al., 2025). EVs derived from the gut microbiota have recently emerged as critical mediators of microbiota-host communication in bone diseases (Jones et al., 2018). By crossing the intestinal barrier and entering systemic circulation, GM-EVs influence distant skeletal tissues through immune, metabolic, and endocrine pathways (Chen et al., 2022).

Beneficial GM-EVs promote OB activity, suppress osteoclastogenesis, and preserve bone mass, whereas EVs derived from pathogenic or dysbiotic bacteria can induce inflammation, disrupt immune balance, and accelerate bone loss (Liu et al., 2021; Chen et al., 2022; Wang et al., 2022; Bielaszewska et al., 2018). For instance, EVs from the beneficial bacterium *Akkermansia muciniphila* accumulate in bone tissue, promoting mineralization and inhibiting OC, which improves bone density in ovariectomized mice. Conversely, reduced abundance of beneficial bacteria diminishes these protective effects (Liu et al., 2021). GM-EVs regulate bone health by modulating inflammatory signaling (e.g., TLR-NF-κB) and immune cell differentiation (e.g., Treg/Th17 balance). EVs from the probiotic *Lactobacillus johnsonii* inhibit mTORC1 and promote anti-inflammatory M2 macrophage polarization, thereby alleviating OA-associated cartilage damage (Liu R. et al., 2023). In RA, EVs from the probiotic *Propionibacterium freudenreichii* mitigate RA by reducing collagen-specific antibodies, suppressing pro-inflammatory cytokines, and inhibiting OC formation and bone erosion (Woo et al., 2024). In contrast, *Fusobacterium nucleatum* EVs deliver the virulence factor FadA to synovial macrophages, activating the Rab5a/YB-1 axis to drive IL-6 and TNF-α production and tissue destruction (Hong et al., 2023). They are also involved in metabolic pathways mediated by short-chain fatty acids (SCFAs), serotonin, bile acids, and trimethylamine N-oxide (TMAO) (Peng et al., 2021; Yang et al., 2024; Hu et al., 2022).

Beyond direct skeletal effects, GM-EVs maintain intestinal barrier integrity and shape microbial ecology, thereby preventing systemic inflammation that contributes to bone degeneration (Thoo et al., 2019; Ciccia et al., 2017; Li et al., 2019; Liang et al., 2022). Intestinal dysbiosis and barrier dysfunction allow the translocation of pathogens and toxins into the systemic circulation, affecting distal tissues including bone (Yan et al., 2016; Guido et al., 2021). *Lactobacillus fermentum* EVs increase microbial diversity and the abundance of beneficial genera (Gao et al., 2026). *Akkermansia muciniphila* EVs facilitate intraspecies competition by inhibiting rival clades and modulating host immunity to reinforce niche dominance (Hong et al., 2025). Furthermore, EVs from probiotic strains like *E. coli Nissle 1917* (EcN) enhance tight junction proteins (ZO-1, claudin-14), increase transepithelial electrical resistance (TEER), and induce anti-inflammatory cytokines and antimicrobial peptides (hBD-2), thereby strengthening the barrier (Alvarez et al., 2016). Emerging evidence further suggests that GM-EVs participate in multi-organ communication within the “brain-gut-bone axis”, offering a novel paradigm for systemic regulation of skeletal homeostasis (Li R. et al., 2024; Zhang YW. et al., 2021; Bravo et al., 2011; Siva Venkatesh et al., 2024). The “brain-gut-bone axis” is a complex, integrated network linking the central nervous system (CNS), gut microbiota, and skeletal system via multi-directional communication, involving neural, immune, endocrine, and metabolic pathways (Li R. et al., 2024; Zhang YW. et al., 2021). However, when evaluating this complex cross-talk, it is pivotal to distinguish clinical observational correlations from mechanistically proven causalities.

Gut-Brain Interactions: Clinical studies often report associations between intestinal dysbiosis and neurological disorders, such as the co-occurrence of depression and inflammatory bowel disease (Huang X. et al., 2021). Beyond these correlations, experimental models provide evidence for the causal relationships in this bi-directional communication. For instance, *Lactobacillus rhamnosus* JB-1 modulates neurobehavior via vagal pathways (Bravo et al., 2011). This specific link is supported by vagotomy models, in which severing the vagus nerve prevents these microbe-induced behavioral changes (Sgritta et al., 2019). Conversely, the brain influences gut microbiota through sympathetic nerve signaling to intestinal immune cells, protecting against dysbiosis (Gabanyi et al., 2016). Moreover, direct intervention studies have revealed the effects of microbial metabolites: exogenous supplementation with SCFAs attenuates neuroinflammation and regulates brain function (Siva Venkatesh et al., 2024).

Brain-Bone Interactions: Epidemiological studies have long established a positive correlation between chronic psychological stress (or depression) and decreased bone mineral density (Zhang YW. et al., 2021). Beyond these correlations, experimental models further reveal the causal mechanisms underlying this interaction. Specifically, the central nervous system regulates bone metabolism via specific neurotransmitters (e.g., norepinephrine, neuropeptide Y). Mice deficient in sympathetic β2-adrenergic receptors exhibit increased bone mass, demonstrating a causal inhibitory effect of sympathetic tone on OB activity (Otto et al., 2020). Reciprocally, bone functions as an endocrine organ. Direct administration of bone-derived mediators, such as osteocalcin (OCN) and FGF18, alleviates motor deficits in parkinson’s models, indicating a functional bone-to-brain feedback loop (Guo et al., 2018; Guo et al., 2017).

**FIGURE 3:**
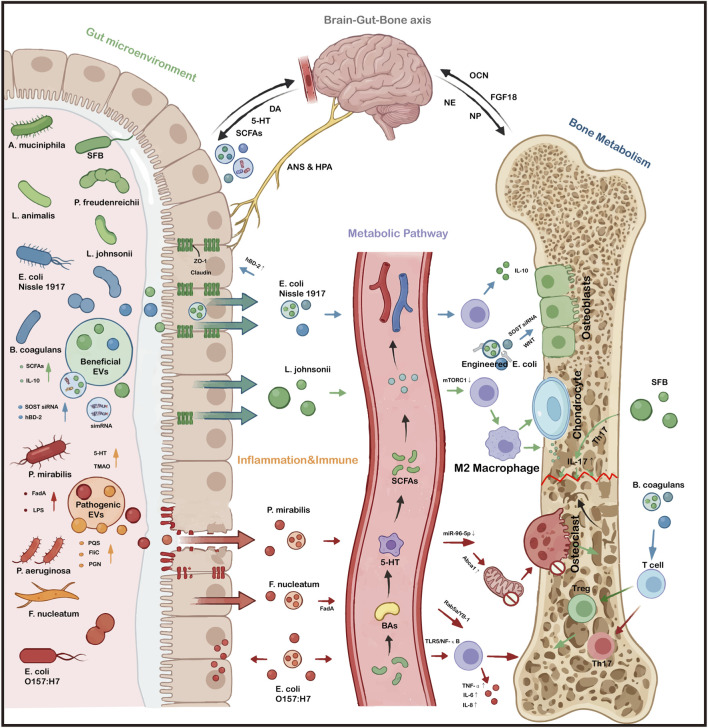
GM-EVs in the Regulation of Bone Homeostasis. In summary, this figure elucidates the complex multi-organ crosstalk within the “brain-gut-bone axis”, revealing how beneficial and pathogenic GM-EVs bidirectionally dictate bone remodeling by systematically integrating gut-derived metabolites (e.g., SCFAs, 5-HT, TMAO), immune responses, and central neuroendocrine signaling pathways.

As previously detailed, the “gut-bone axis” highlights the gut’s influence on skeletal health. These three axes are functionally interlinked, forming a synergistic regulatory network where each component acts as both a “signal source” and “target effector”. Intestinal microbiota-derived EVs are crucial carriers for precise inter-organ signal transfer within this “brain-gut-bone axis”. Their protective membrane structure allows them to traverse physiological barriers like the intestinal mucosa and blood-brain barrier, overcoming issues of signal degradation and poor targeting seen with free metabolites. For instance, *Lactobacillus* EVs promote neurite growth and neuroprotection more effectively and stably than free metabolites in alzheimer’s models (Kim et al., 2024). Although mechanistic understanding and safety profiling remain incomplete, existing research has shown that the primary safety concerns associated with GM-EVs stem from the pathogen-associated molecular patterns (PAMPs) they carry, particularly lipopolysaccharides (LPS) derived from the outer membranes of Gram-negative bacteria (Chen et al., 2025). Given that the gut microbiota is a complex ecosystem composed of both beneficial bacteria and potentially pathogenic bacteria, GM-EV preparations may inevitably contain vesicles derived from Gram-negative pathogenic commensals with high LPS loads. When exposed in their entirety, there is a risk of inducing systemic inflammation or localized excessive inflammation (Broz, 2016). It is important to note that this risk is strain-dependent. Therefore, rigorous LPS removal or strain screening is essential to ensure the safety of GM-EVs therapy (Liang et al., 2025). Furthermore, caution is warranted when translating GM-EVs therapy into clinical practice. First, most of the current insights into the underlying mechanisms are derived from animal models, whose microbiome composition and immune responses differ significantly from those of humans. Second, animal studies typically use high concentrations of GM-EVs isolated *in vitro*, which may not accurately reflect the physiological doses that cross the human intestinal barrier. Finally, given the highly individualized nature of the human gut microbiome, the universality of the efficacy of single-strain GM-EV interventions remains a major challenge to be addressed in future clinical trials. GM-EVs represent a promising therapeutic avenue for metabolic and inflammatory bone disorders (as summarized in Figure 3; Table 3). It must be emphasized that direct experimental evidence demonstrating that GM-EVs regulate bone specifically through the CNS is currently lacking. Most mechanistic claims regarding this multi-organ axis are speculative and inferred from separate “gut-brain axis”, “brain-bone axis ”, and “gut-bone axis”. However, based on the established individual axes, an indirect “gut-brain-bone” pathway is mechanistically plausible: GM-EVs may initially modulate CNS function, which subsequently transmits altered neural signals to regulate bone metabolism (Li R. et al., 2024). Although an indirect “gut-brain-bone” pathway is mechanistically plausible, rigorous *in vivo* tracing and targeted knockout models are required to definitively prove this causal link. Furthermore, validating this multi-organ axis warrants further research to identify specific EVs cargos and their downstream neural targets.

**TABLE 3:** Summary of key cargo molecules in GM-EVs and their functions and regulatory mechanisms in skeletal diseases.

EVs type	EVs source	Molecular mediators in the EVs	Function and mechanism of action	References
GM-EVs GM-EVs	*Akkermansia muciniphila*	-	Promotes bone mineralization and inhibits osteoclast activity to improve bone density	Liu et al. (2021)
	*Lactobacillus animalis*	-	Promotes bone formation/angiogenesis and inhibits apoptosis to alleviate hormone-induced osteonecrosis	Chen et al. (2022)
	*Lactobacillus johnsonii*	-	Inhibits mTORC1 to promote M2 macrophage polarization and ameliorate OA	Liu R et al. (2023)
	*Escherichia coli*	SOST siRNA	Activates WNT pathway to promote OB differentiation and increase bone density	Liu H et al. (2023)
	*E. coli Nissle 1917*	-	Increases IL-10, reduces pro-inflammatory cytokines, and enhances tight junction proteins/TEER/hBD-2 to strengthen intestinal barrier	Alvarez et al. (2016)
	*E. coli O157:H7*	Flagellin	Activates NF-κB via TLR5 to induce IL-8 production and intestinal inflammation	Bielaszewska et al. (2018)
	*Propionibacterium freudenreichii*	-	Inhibits pro-inflammatory factors and OCs formation to alleviat RA	Woo et al. (2024)
	*Proteus mirabilis*	-	Downregulates miR-96-5p and upregulates Abca1 to induce OC apoptosis and mitochondrial dysfunction, improving OP	Wang et al. (2022)
	*Fusobacterium nucleatum*	FadA	Activates Rab5a/YB-1 to promote IL-6/TNF-α and exacerbate OA; activates RIPK1 to induce oxidative stress and epithelial necroptosis, disrupting intestinal barrier	Hong et al. (2023)
	*Pseudomonas aeruginosa*	Quorum-sensing molecules (e.g., PQS)	Participates in metabolic exchange and disrupts intestinal barrier integrity	Liang et al. (2022)

mTORC1, Mammalian Target of Rapamycin Complex 1; OA, Osteoarthritis; SOST siRNA, Sclerostin Small Interfering RNA; OB, Osteoblast; TEER, Transepithelial Electrical Resistance; hBD-2, Human Beta-Defensin 2; TLR5, Toll-Like Receptor 5; OCs, Osteoclasts; RA, Rheumatoid Arthritis; Abca1, ATP-Binding Cassette Subfamily A Member 1; OP, Osteoporosis; FadA, *Fusobacterium* Adhesin A; PQS, *Pseudomonas* Quinolone Signal.

## 5 Comparison of EVs from different sources

EVs derived from mammalian cells, plants, and the gut microbiota share a common role as carriers of bioactive signals but differ substantially in origin, mechanism of action, and therapeutic applicability (as summarized in Table 4). MEVs offer high targeting precision and direct regenerative capacity, making them suitable for personalized and localised bone repair (Garcia et al., 2016; Kim et al., 2026; Zou et al., 2023). PDEVs provide scalable, low-toxicity, and pleiotropic anti-inflammatory and antioxidant effects, supporting their use as adjunctive therapies (Zeng et al., 2024; Zhang et al., 2025). GM-EVs uniquely exploit cross-system regulation via the “gut-bone axis”, enabling indirect but sustained modulation of systemic bone homeostasis (Liu H. et al., 2023; Cheung et al., 2022; Lu et al., 2025; Zu et al., 2024).

**TABLE 4:** Comparative analysis of mammalian-derived, plant-derived, and gut microbiota-derived EVs in bone health regulation.

EVs source	Key advantages	Limitations	Applicable bone disorders	References
MEVs	Low immunogenicity & High biocompatibility Natural “homing” ability to injury sites High potential for personalized engineering	Low natural yield & High cost Isolation/purification is non-standardized and difficult	Chronic & Localized repair: OA, Chronic bone defects, OP, Bone tumors (Less practical for acute conditions)	Yin et al. (2022), Thoo et al. (2019), Li R et al. (2024)
PDEVs	High yield, scalable, & cost-effective Low immunogenicity and no ethical concerns High GI stability (suitable for oral delivery) Intrinsic bioactivity (no loading needed)	Low targeting specificity High batch-to-batch variation Undefined surface markers and isolation protocols	Multi-mechanistic & Adjunctive therapy: OA, Rotator cuff injuries, OP (Unclear potential for large defects/tumors)	Liu et al. (2017), Lian et al. (2022), Zhang Y. W. et al. (2021)
GM-EVs	Systemic regulation via “gut-bone axis” High oral bioavailability Strain specificity High adaptability for drug loading	Potential toxicity/pathogenicity Bioavailability and absorption pathways are poorly understood Lack of unified isolation protocols	Systemic & Metabolic Bone Diseases: OP (gut dysbiosis linked), Steroid-induced osteonecrosis, Early OA, Inflammatory Bowel Disease-associated bone loss, Autoimmune bone diseases (e.g., RA)	Liu C et al. (2022), Liu et al. (2021), Chen et al. (2022), Bravo et al. (2011), Siva Venkatesh et al. (2024), Huang X et al. (2021), Sgritta et al. (2019), Gabanyi et al. (2016)

GI, gastrointestinal; OA, osteoarthritis; OP, osteoporosis; RA, rheumatoid arthritis.

Currently, the main methods for isolating EVs include ultracentrifugation (UC), density gradient centrifugation (DGC), size-exclusion chromatography (SEC), tangential flow filtration (TFF), and immunoaffinity capture (Welsh et al., 2024). However, methods for isolating EVs vary depending on their source. MEVs are typically isolated using established UC, SEC, TFF protocols, which are relatively well-standardized (Auquière et al., 2025; Aliakbari et al., 2024). PDEVs require additional purification steps to remove interfering substances such as cellulose and pectin, often involving a combination of SEC and UC (Lv et al., 2024; Bokka et al., 2020). The isolation of GM-EVs poses the greatest challenge: these vesicles must be isolated from complex fecal matrices or culture media rich in heterologous proteins and LPS, and typically require a combination of DGC and ultrafiltration to achieve sufficient purity (Wu et al., 2024; Northrop-Albrecht et al., 2022).

In addition, clinical translation requires selecting the appropriate route of administration based on the specific type of EVs. EVs can be administered via various routes, including intravenous, intraperitoneal, oral and local administration (such as intratumoral or intra-articular injection) (Su et al., 2025). In the treatment of skeletal disorders, MEVs can be delivered via local intra-articular injection or scaffold implantation to maximize local retention and minimize systemic clearance (Li et al., 2025; Huang and Xie, 2025). Due to their gastrointestinal stability, PDEVs are suitable for oral administration. Research evidence suggests that PDEVs can regulate intestinal homeostasis and the gut microbiota, which may enable non-invasive delivery via the “gut-bone axis” (Zhan et al., 2026; Guan et al., 2025). GM-EVs, particularly those derived from probiotics, naturally possess the ability to cross the intestinal barrier and are therefore equally suitable for oral administration (Liang et al., 2025; Wang K. et al., 2025). Although intravenous injection is widely used, EVs primarily accumulate in organs such as the liver, lungs and spleen, and their half-life is relatively short (Su et al., 2025). Therefore, choosing the appropriate route of administration is crucial.

Future translation of EVs-based therapies will require standardized isolation and characterization protocols, improved understanding of *in vivo* biodistribution and targeting, and advanced engineering strategies to enhance safety and efficacy. Integrating synthetic biology, materials science, and precision medicine approaches will be essential to unlock the full therapeutic potential of EVs in bone diseases.

## 6 Conclusion

EVs derived from mammalian cells, plants, and the gut microbiota represent a versatile and biologically compatible therapeutic platform for bone diseases. Despite their shared function as carriers of bioactive molecules, these EVs operate through distinct regulatory paradigms. MEVs primarily enable local, high-precision modulation of bone metabolism, inflammation, and tissue regeneration. PDEVs exert pleiotropic anti-inflammatory and antioxidant effects that improve the bone microenvironment and support repair processes. GM-EVs uniquely integrate immune, metabolic, and microbial signals through the “gut-bone axis”, offering a systemic and cross-organ approach to skeletal regulation.

Together, these complementary EVs sources establish a unified framework for EVs-based precision therapy in bone disorders, ranging from local degenerative diseases to systemic metabolic and inflammatory conditions. However, significant challenges remain, including the lack of standardized isolation and characterization methods, incomplete understanding of *in vivo* biodistribution and targeting, and unresolved safety concerns, particularly for microbiota-derived EVs. In addition, the long-term storage stability of EVs remains a significant barrier to clinical translation. Repeated freeze-thaw cycles and improper storage conditions can compromise vesicle integrity, reduce particle concentration, and diminish biological activity (Ahmadian et al., 2024).

To overcome the aforementioned limitations in biodistribution and therapeutic efficacy, engineered EVs have been actively developed and investigated as a promising strategy (Malekian et al., 2023). Current modification strategies primarily involve surface modification, cargo loading and targeting peptides. Surface modification can be achieved via before-separation modification (e.g., genetic engineering, metabolic engineering, and direct parent cell membrane engineering) or post-separation modification (e.g., physical and chemical modifications). Before-separation modification preserves native EVs structure but requires complex cell engineering and suffers from batch variability, whereas post-separation modification offers greater flexibility but may compromise EVs integrity and surface functionality (Hu et al., 2025). For cargo loading, exogenous methods (e.g., electroporation, sonication, freeze-thaw, extrusion) are widely used; among them, freeze-thaw and hypotonic dialysis show superior loading efficiency for macromolecules like mRNA and proteins, yet all exogenous techniques face significant cargo loss, low reproducibility, and potential damage to EVs membranes. Endogenous loading via producer cells maintains EVs naturalness but offers poor controllability and is unsuitable for synthetic drugs (Zhan H et al., 2023; Mendonca et al., 2025). Targeting peptides involve displaying specific short peptides on the surface of EVs, enabling them to actively recognize and accumulate at lesion sites. Depending on the EVs membrane proteins utilized, a diverse array of targeting peptides can be displayed (e.g., RGD, GE11, RVG, iRGD). While peptides are low in immunogenicity and easy to synthesize, their optimal conformation, surface density, and *in vivo* stability remain challenging, and improper conjugation may impair EVs targeting (Song et al., 2022).

Future progress will depend on elucidating source-specific mechanisms, identifying key functional cargoes, and optimizing EVs engineering strategies to enhance targeting accuracy and therapeutic efficacy. However, translating these mechanistic insights into clinical practice requires overcoming key translational bottlenecks, particularly the need for rigorous standardized protocols for EVs isolation, characterization, and storage, as well as the development of source-specific delivery strategies. In addition, advances at the intersection of extracellular vesicle biology, synthetic biology, and materials science are expected to accelerate clinical translation, positioning EVs-based therapies as a next-generation strategy for the treatment of bone diseases.

## References

[B1] Ahmadian S. Jafari N. Tamadon A. Ghaffarzadeh A. Rahbarghazi R. Mahdipour M. (2024). Different storage and freezing protocols for extracellular vesicles: a systematic review. Stem Cell Res. Ther. 15 (1), 453. 10.1186/s13287-024-04005-7 39593194 PMC11600612

[B2] Aliakbari F. Stocek N. B. Cole-André M. Gomes J. Fanchini G. Pasternak S. H. (2024). A methodological primer of extracellular vesicles isolation and characterization via different techniques. Biol. Methods Protoc. 9 (1), bpae009. 10.1093/biomethods/bpae009 38425334 PMC10902684

[B3] Alvarez C. S. Badia J. Bosch M. Giménez R. Baldomà L. (2016). Outer membrane vesicles and soluble factors released by probiotic *Escherichia coli* Nissle 1917 and commensal ECOR63 enhance barrier function by regulating expression of tight junction proteins in intestinal epithelial cells. Front. Microbiol. 7, 1981. 10.3389/fmicb.2016.01981 28018313 PMC5156689

[B4] Auquière M. Muccioli G. G. des Rieux A. (2025). Methods and challenges in purifying drug-loaded extracellular vesicles. J. Extracell. Vesicles 14 (6), e70097. 10.1002/jev2.70097 40527729 PMC12173533

[B5] Ayers C. Kansagara D. Lazur B. Fu R. Kwon A. Harrod C. (2023). Effectiveness and safety of treatments to prevent fractures in people with low bone mass or primary osteoporosis: a living systematic review and network meta-analysis for the American college of physicians. Ann. Intern Med. 176 (2), 182–195. 10.7326/m22-0684 36592455

[B6] Bellido T. (2024). Bisphosphonates for osteoporosis: from bench to clinic. J. Clin. Invest 134 (6), e179942. 10.1172/jci179942 38488010 PMC10940084

[B7] Bielaszewska M. Marejková M. Bauwens A. Kunsmann-Prokscha L. Mellmann A. Karch H. (2018). Enterohemorrhagic *Escherichia coli* O157 outer membrane vesicles induce interleukin 8 production in human intestinal epithelial cells by signaling via toll-like receptors TLR4 and TLR5 and activation of the nuclear factor NF-κB. Int. J. Med. Microbiol. 308 (7), 882–889. 10.1016/j.ijmm.2018.06.004 29934223

[B8] Bokka R. Ramos A. P. Fiume I. Manno M. Raccosta S. Turiák L. (2020). Biomanufacturing of tomato-derived nanovesicles. Foods 9 (12), 1852. 10.3390/foods9121852 33322632 PMC7764365

[B9] Bravo J. A. Forsythe P. Chew M. V. Escaravage E. Savignac H. M. Dinan T. G. (2011). Ingestion of Lactobacillus strain regulates emotional behavior and central GABA receptor expression in a mouse via the vagus nerve. Proc. Natl. Acad. Sci. U. S. A. 108 (38), 16050–16055. 10.1073/pnas.1102999108 21876150 PMC3179073

[B10] Broz P. (2016). Inflammasomes: intracellular detection of extracellular bacteria. Cell Res. 26 (8), 859–860. 10.1038/cr.2016.67 27283800 PMC4973328

[B11] Cao Y. Tan X. Shen J. Liu F. Xu Y. Chen Y. (2024). Morinda Officinalis-derived extracellular vesicle-like particles: anti-osteoporosis effect by regulating MAPK signaling pathway. Phytomedicine 129, 155628. 10.1016/j.phymed.2024.155628 38663117

[B12] Chen C. Y. Rao S. S. Yue T. Tan Y. J. Yin H. Chen L. J. (2022). Glucocorticoid-induced loss of beneficial gut bacterial extracellular vesicles is associated with the pathogenesis of osteonecrosis. Sci. Adv. 8 (15), eabg8335. 10.1126/sciadv.abg8335 35417243 PMC9007505

[B13] Chen J. Shi X. Deng Y. Dang J. Liu Y. Zhao J. (2024). miRNA-148a-containing GMSC-derived EVs modulate Treg/Th17 balance via IKKB/NF-κB pathway and treat a rheumatoid arthritis model. JCI Insight 9 (10), e177841. 10.1172/jci.insight.177841 38652539 PMC11141912

[B14] Chen M. Y. Cheng T. W. Pan Y. C. Mou C. Y. Chiang Y. W. Lin W. C. (2025). Endotoxin-free outer membrane vesicles for safe and modular anticancer immunotherapy. ACS Synth. Biol. 14 (1), 148–160. 10.1021/acssynbio.4c00483 39763210 PMC11744915

[B15] Cheung K. C. P. Jiao M. Xingxuan C. Wei J. (2022). Extracellular vesicles derived from host and gut microbiota as promising nanocarriers for targeted therapy in osteoporosis and osteoarthritis. Front. Pharmacol. 13, 1051134. 10.3389/fphar.2022.1051134 36686680 PMC9859449

[B16] Chew J. R. J. Chuah S. J. Teo K. Y. W. Zhang S. Lai R. C. Fu J. H. (2019). Mesenchymal stem cell exosomes enhance periodontal ligament cell functions and promote periodontal regeneration. Acta Biomater. 89, 252–264. 10.1016/j.actbio.2019.03.021 30878447

[B17] Ciccia F. Guggino G. Rizzo A. Alessandro R. Luchetti M. M. Milling S. (2017). Dysbiosis and zonulin upregulation alter gut epithelial and vascular barriers in patients with ankylosing spondylitis. Ann. Rheum. Dis. 76 (6), 1123–1132. 10.1136/annrheumdis-2016-210000 28069576 PMC6599509

[B18] Cui Y. Gao J. He Y. Jiang L. (2020). Plant extracellular vesicles. Protoplasma 257 (1), 3–12. 10.1007/s00709-019-01435-6 31468195

[B19] Davies O. G. Cox S. C. Williams R. L. Tsaroucha D. Dorrepaal R. M. Lewis M. P. (2017). Annexin-enriched osteoblast-derived vesicles act as an extracellular site of mineral nucleation within developing stem cell cultures. Sci. Rep. 7 (1), 12639. 10.1038/s41598-017-13027-6 28974747 PMC5626761

[B20] De Robertis M. Sarra A. D'Oria V. Mura F. Bordi F. Postorino P. (2020). Blueberry-derived exosome-like nanoparticles counter the response to TNF-α-Induced change on gene expression in EA.hy926 cells. Biomolecules 10 (5), 742. 10.3390/biom10050742 32397678 PMC7277966

[B21] Depypere M. Morgenstern M. Kuehl R. Senneville E. Moriarty T. F. Obremskey W. T. (2020). Pathogenesis and management of fracture-related infection. Clin. Microbiol. Infect. 26 (5), 572–578. 10.1016/j.cmi.2019.08.006 31446152

[B22] Du Y. Liu Y. Zhang Y. Nie Y. Xu Z. Qin L. (2025). Structurally and functionally adaptive biomimetic periosteum: materials, fabrication, and construction strategies. Explor. (Beijing) 5 (3), 70005. 10.1002/exp.70005 40585765 PMC12199452

[B23] Fan Y. Pedersen O. (2021). Gut microbiota in human metabolic health and disease. Nat. Rev. Microbiol. 19 (1), 55–71. 10.1038/s41579-020-0433-9 32887946

[B24] Fang F. Yang J. Wang J. Li T. Wang E. Zhang D. (2024). The role and applications of extracellular vesicles in osteoporosis. Bone Res. 12 (1), 4. 10.1038/s41413-023-00313-5 38263267 PMC10806231

[B25] Gabanyi I. Muller P. A. Feighery L. Oliveira T. Y. Costa-Pinto F. A. Mucida D. (2016). Neuro-immune interactions drive tissue programming in intestinal macrophages. Cell 164 (3), 378–391. 10.1016/j.cell.2015.12.023 26777404 PMC4733406

[B26] Gao C. Zhao W. Feng R. Zhang L. Ge L. Sun J. (2026). Melanin nanoparticles-loaded lactobacillus fermentum exosomes for targeted and visualized treatment of ulcerative colitis. J. Adv. Res. 82, 1049–1067. 10.1016/j.jare.2025.06.068 40555281 PMC13001177

[B27] Garcia J. Wright K. Roberts S. Kuiper J. H. Mangham C. Richardson J. (2016). Characterisation of synovial fluid and infrapatellar fat pad derived mesenchymal stromal cells: the influence of tissue source and inflammatory stimulus. Sci. Rep. 6, 24295. 10.1038/srep24295 27073003 PMC4829842

[B28] GBD 2021 Diseases and Injuries Collaborators (2024). Global incidence, prevalence, years lived with disability (YLDs), disability-adjusted life-years (DALYs), and healthy life expectancy (HALE) for 371 diseases and injuries in 204 countries and territories and 811 subnational locations, 1990-2021: a systematic analysis for the Global burden of disease study 2021. Lancet 403 (10440), 2133–2161. 10.1016/s0140-6736(24)00757-8 38642570 PMC11122111

[B29] GBD 2021 Other Musculoskeletal Disorders Collaborators (2023). Global, regional, and national burden of other musculoskeletal disorders, 1990-2020, and projections to 2050: a systematic analysis of the Global burden of disease study 2021. Lancet Rheumatol. 5 (11), e670–e682. 10.1016/s2665-9913(23)00232-1 37927903 PMC10620749

[B30] Giancaterino S. Boi C. (2023). Alternative biological sources for extracellular vesicles production and purification strategies for process scale-up. Biotechnol. Adv. 63, 108092. 10.1016/j.biotechadv.2022.108092 36608746

[B31] Guan X. Zhu M. Zhu H. Wang Q. Chen J. Chen Y. (2025). Oral natural extracellular vesicles for biomedical applications: advances and clinical perspectives. J. Adv. Res. 83, 1105–1125. 10.1016/j.jare.2025.08.003 40783070 PMC13131399

[B32] Guido G. Ausenda G. Iascone V. Chisari E. (2021). Gut permeability and osteoarthritis, towards a mechanistic understanding of the pathogenesis: a systematic review. Ann. Med. 53 (1), 2380–2390. 10.1080/07853890.2021.2014557 34933614 PMC8725942

[B33] Guo X. Liu T. Zhao D. Wang X. Liu D. He Y. (2017). FGF18 protects against 6-hydroxydopamine-induced nigrostriatal damage in a rat model of Parkinson's disease. Neuroscience 356, 229–241. 10.1016/j.neuroscience.2017.05.007 28504195

[B34] Guo X. Z. Shan C. Hou Y. F. Zhu G. Tao B. Sun L. H. (2018). Osteocalcin ameliorates motor dysfunction in a 6-Hydroxydopamine-Induced Parkinson's disease rat model through AKT/GSK3β signaling. Front. Mol. Neurosci. 11, 343. 10.3389/fnmol.2018.00343 30319352 PMC6170617

[B35] Hong M. Li Z. Liu H. Zheng S. Zhang F. Zhu J. (2023). Fusobacterium nucleatum aggravates rheumatoid arthritis through FadA-containing outer membrane vesicles. Cell Host Microbe 31 (5), 798–810.e7. 10.1016/j.chom.2023.03.018 37054714

[B36] Hong M. G. Song E. J. Yoon H. J. Chung W. H. Seo H. Y. Kim D. (2025). Clade-specific extracellular vesicles from Akkermansia muciniphila mediate competitive colonization via direct inhibition and immune stimulation. Nat. Commun. 16 (1), 2708. 10.1038/s41467-025-57631-x 40108178 PMC11923206

[B37] Hu W. Cai C. Li Y. Kang F. Chu T. Dong S. (2022). Farnesoid X receptor agonist attenuates subchondral bone osteoclast fusion and osteochondral pathologies of osteoarthritis via suppressing JNK1/2/NFATc1 pathway. Faseb J. 36 (4), e22243. 10.1096/fj.202101717R 35224782

[B38] Hu M. Han Y. Zhang X. Tian S. Shang Z. Yuan Z. (2025). Extracellular vesicles for targeted drug delivery: advances in surface modification strategies and therapeutic applications. J. Transl. Med. 23 (1), 1028. 10.1186/s12967-025-07077-y 41029680 PMC12486671

[B39] Huang Y. Xie H. (2025). Extracellular vesicle-integrated biomaterials in bone tissue engineering applications: current progress and future perspectives. Int. J. Nanomedicine 20, 7653–7683. 10.2147/ijn.S522198 40546799 PMC12182063

[B40] Huang Y. Zhang X. Zhan J. Yan Z. Chen D. Xue X. (2021). Bone marrow mesenchymal stem cell-derived exosomal miR-206 promotes osteoblast proliferation and differentiation in osteoarthritis by reducing Elf3. J. Cell Mol. Med. 25 (16), 7734–7745. 10.1111/jcmm.16654 34160894 PMC8358849

[B41] Huang X. Hussain B. Chang J. (2021). Peripheral inflammation and blood-brain barrier disruption: effects and mechanisms. CNS Neurosci. Ther. 27 (1), 36–47. 10.1111/cns.13569 33381913 PMC7804893

[B42] Huang J. Wang X. Wang Z. Deng L. Wang Y. Tang Y. (2023). Extracellular vesicles as a novel mediator of interkingdom communication. Cytokine Growth Factor Rev. 73, 173–184. 10.1016/j.cytogfr.2023.08.005 37634980

[B43] Hwang J. H. Park Y. S. Kim H. S. Kim D. H. Lee S. H. Lee C. H. (2023). Yam-derived exosome-like nanovesicles stimulate osteoblast formation and prevent osteoporosis in mice. J. Control Release 355, 184–198. 10.1016/j.jconrel.2023.01.071 36736431

[B44] Jiao Q. Liu J. Zhou L. McClements D. J. Liu W. Luo J. (2025). Lactobacillus extracellular vesicles alleviate alcohol-induced liver injury in mice by regulating gut microbiota and activating the Nrf-2 signaling pathway. Food Funct. 16 (4), 1284–1298. 10.1039/d4fo04364b 39865864

[B45] Jones R. M. Mulle J. G. Pacifici R. (2018). Osteomicrobiology: the influence of gut microbiota on bone in health and disease. Bone 115, 59–67. 10.1016/j.bone.2017.04.009 28433758

[B46] Kim M. Park J. H. (2022). Isolation of aloe saponaria-Derived extracellular vesicles and investigation of their potential for chronic wound healing. Pharmaceutics 14 (9), 1905. 10.3390/pharmaceutics14091905 36145653 PMC9504946

[B47] Kim J. Zhang S. Zhu Y. Wang R. Wang J. (2023). Amelioration of colitis progression by ginseng-derived exosome-like nanoparticles through suppression of inflammatory cytokines. J. Ginseng Res. 47 (5), 627–637. 10.1016/j.jgr.2023.01.004 37720571 PMC10499592

[B48] Kim N. Y. Lee H. Y. Choi Y. Y. Mo S. J. Jeon S. Ha J. H. (2024). Effect of gut microbiota-derived metabolites and extracellular vesicles on neurodegenerative disease in a gut-brain axis chip. Nano Converg. 11 (1), 7. 10.1186/s40580-024-00413-w 38340254 PMC10858859

[B49] Kim J. Hwang Y. H. Nam G.-H. Kim I.-S. (2026). Breaking barriers: engineering extracellular vesicles for enhanced endosomal escape and therapeutic delivery. J. Control. Release 389, 114462. 10.1016/j.jconrel.2025.114462 41285243

[B50] Kuang M. J. Huang Y. Zhao X. G. Zhang R. Ma J. X. Wang D. C. (2019). Exosomes derived from Wharton's jelly of human umbilical cord mesenchymal stem cells reduce osteocyte apoptosis in glucocorticoid-induced osteonecrosis of the femoral head in rats via the miR-21-PTEN-AKT signalling pathway. Int. J. Biol. Sci. 15 (9), 1861–1871. 10.7150/ijbs.32262 31523188 PMC6743291

[B51] Kumar M. A. Baba S. K. Sadida H. Q. Marzooqi S. A. Jerobin J. Altemani F. H. (2024). Extracellular vesicles as tools and targets in therapy for diseases. Signal Transduct. Target Ther. 9 (1), 27. 10.1038/s41392-024-01735-1 38311623 PMC10838959

[B52] Li C. Huang Q. Yang R. Dai Y. Zeng Y. Tao L. (2019). Gut microbiota composition and bone mineral loss-epidemiologic evidence from individuals in Wuhan, China. Osteoporos. Int. 30 (5), 1003–1013. 10.1007/s00198-019-04855-5 30666372

[B53] Li K. Yan G. Huang H. Zheng M. Ma K. Cui X. (2022). Anti-inflammatory and immunomodulatory effects of the extracellular vesicles derived from human umbilical cord mesenchymal stem cells on osteoarthritis via M2 macrophages. J. Nanobiotechnology 20 (1), 38. 10.1186/s12951-021-01236-1 35057811 PMC8771624

[B54] Li W. Li L. Cui R. Chen X. Hu H. Qiu Y. (2023). Bone marrow mesenchymal stem cells derived exosomal Lnc TUG1 promotes bone fracture recovery via miR-22-5p/Anxa8 axis. Hum. Cell 36 (3), 1041–1053. 10.1007/s13577-023-00881-y 36952210 PMC10110643

[B55] Li Y. Yue G. Yu S. Liu Z. Cao Y. Wang X. (2024). Extracellular vesicles derived from H(2)O(2)-Stimulated adipose-derived stem cells alleviate senescence in diabetic bone marrow mesenchymal stem cells and restore their osteogenic capacity. Drug Des. Devel Ther. 18, 2103–2124. 10.2147/dddt.S454509 38882044 PMC11177868

[B56] Li R. Miao Z. Liu Y. Chen X. Wang H. Su J. (2024). The brain-gut-bone axis in neurodegenerative diseases: insights, challenges, and future prospects. Adv. Sci. (Weinh) 11 (38), e2307971. 10.1002/advs.202307971 39120490 PMC11481201

[B57] Li Y. Fu T. Yu W. Wen H. Wang Z. Lyu Z. (2025). Mesenchymal stem cells and extracellular vesicles for knee osteoarthritis: clinical application, mechanism exploration and prospect. Stem Cell Res. Ther. 16 (1), 688. 10.1186/s13287-025-04783-8 41276868 PMC12751560

[B58] Lian M. Q. Chng W. H. Liang J. Yeo H. Q. Lee C. K. Belaid M. (2022). Plant-derived extracellular vesicles: recent advancements and current challenges on their use for biomedical applications. J. Extracell. Vesicles 11 (12), e12283. 10.1002/jev2.12283 36519808 PMC9753580

[B59] Liang X. Dai N. Sheng K. Lu H. Wang J. Chen L. (2022). Gut bacterial extracellular vesicles: important players in regulating intestinal microenvironment. Gut Microbes 14 (1), 2134689. 10.1080/19490976.2022.2134689 36242585 PMC9578468

[B60] Liang W. Zhou C. Bai J. Zhang H. Long H. Jiang B. (2024). Prospective applications of bioactive materials in orthopedic therapies: a review. Heliyon 10 (16), e36152. 10.1016/j.heliyon.2024.e36152 39247306 PMC11379564

[B61] Liang P. Chen X. Su Z. Luo Y. Wang T. Li J. (2025). The role of oral and gut microbiota in bone health: insights from bacterial extracellular vesicles. Microorganisms 13 (10), 2254. 10.3390/microorganisms13102254 41156714 PMC12566059

[B62] Liu X. Li Q. Niu X. Hu B. Chen S. Song W. (2017). Exosomes secreted from human-induced pluripotent stem cell-derived mesenchymal stem cells prevent osteonecrosis of the femoral head by promoting angiogenesis. Int. J. Biol. Sci. 13 (2), 232–244. 10.7150/ijbs.16951 28255275 PMC5332877

[B63] Liu J. H. Chen C. Y. Liu Z. Z. Luo Z. W. Rao S. S. Jin L. (2021). Extracellular vesicles from child gut microbiota enter into bone to preserve bone mass and strength. Adv. Sci. (Weinh) 8 (9), 2004831. 10.1002/advs.202004831 33977075 PMC8097336

[B64] Liu H. Zhang H. Han Y. Hu Y. Geng Z. Su J. (2022). Bacterial extracellular vesicles-based therapeutic strategies for bone and soft tissue tumors therapy. Theranostics 12 (15), 6576–6594. 10.7150/thno.78034 36185613 PMC9516228

[B65] Liu C. Yan X. Zhang Y. Yang M. Ma Y. Zhang Y. (2022). Oral administration of turmeric-derived exosome-like nanovesicles with anti-inflammatory and pro-resolving bioactions for murine colitis therapy. J. Nanobiotechnology 20 (1), 206. 10.1186/s12951-022-01421-w 35488343 PMC9052603

[B66] Liu Z. Zhuang Y. Fang L. Yuan C. Wang X. Lin K. (2023). Breakthrough of extracellular vesicles in pathogenesis, diagnosis and treatment of osteoarthritis. Bioact. Mater 22, 423–452. 10.1016/j.bioactmat.2022.10.012 36311050 PMC9588998

[B67] Liu R. Zhou Y. Chen H. Xu H. Zuo M. Chen B. (2023). Membrane vesicles from Lactobacillus johnsonii delay osteoarthritis progression via modulating macrophage glutamine synthetase/mTORC1 axis. Biomed. Pharmacother. 165, 115204. 10.1016/j.biopha.2023.115204 37499456

[B68] Liu H. Zhang H. Wang S. Cui J. Weng W. Liu X. (2023). Bone-targeted bioengineered bacterial extracellular vesicles delivering siRNA to ameliorate osteoporosis. Compos. Part B Eng. 255, 110610. 10.1016/j.compositesb.2023.110610

[B69] Liu H. Li R. Yang H. Situ B. Wang G. Xu K. (2025). Extracellular vesicles in gut-bone axis: novel insights and therapeutic opportunities for osteoporosis. Small Sci. 5 (4), 2400474. 10.1002/smsc.202400474 40657203 PMC12244513

[B70] Lou C. Jiang H. Lin Z. Xia T. Wang W. Lin C. (2023). MiR-146b-5p enriched bioinspired exosomes derived from fucoidan-directed induction mesenchymal stem cells protect chondrocytes in osteoarthritis by targeting TRAF6. J. Nanobiotechnology 21 (1), 486. 10.1186/s12951-023-02264-9 38105181 PMC10726686

[B71] Lu J. Yang J. Zheng Y. Chen X. Fang S. (2019). Extracellular vesicles from endothelial progenitor cells prevent steroid-induced osteoporosis by suppressing the ferroptotic pathway in mouse osteoblasts based on bioinformatics evidence. Sci. Rep. 9 (1), 16130. 10.1038/s41598-019-52513-x 31695092 PMC6834614

[B72] Lu J. Wang Y. Wu J. Duan Y. Zhang H. Du H. (2025). Linking microbial communities to rheumatoid arthritis: focus on gut, oral microbiome and their extracellular vesicles. Front. Immunol. 16, 1503474. 10.3389/fimmu.2025.1503474 40308573 PMC12040682

[B73] Lv G. Wang B. Li L. Li Y. Li X. He H. (2022). Exosomes from dysfunctional chondrocytes affect osteoarthritis in sprague-dawley rats through FTO-dependent regulation of PIK3R5 mRNA stability. Bone Jt. Res. 11 (9), 652–668. 10.1302/2046-3758.119.Bjr-2021-0443.R2 36066338 PMC9533253

[B74] Lv L. Li Z. Liu X. Zhang W. Zhang Y. Liang Y. (2024). Revolutionizing medicine: harnessing plant-derived vesicles for therapy and drug transport. Heliyon 10 (22), e40127. 10.1016/j.heliyon.2024.e40127 39634409 PMC11615498

[B75] Malekian F. Shamsian A. Kodam S. P. Ullah M. (2023). Exosome engineering for efficient and targeted drug delivery: current status and future perspective. J. Physiol. 601 (22), 4853–4872. 10.1113/jp282799 35570717

[B76] Mendonca S. R. Bangera P. D. Keerikkadu M. Tippavajhala V. K. Rathnanand M. (2025). Multifunctional engineering of exosomes for precision therapeutics: strategies for targeted delivery, barrier evasion, and clinical translation. Pharm. Res. 42 (11), 1931–1952. 10.1007/s11095-025-03961-w 41188685 PMC12698739

[B77] Ni Z. Kuang L. Chen H. Xie Y. Zhang B. Ouyang J. (2019). The exosome-like vesicles from osteoarthritic chondrocyte enhanced mature IL-1β production of macrophages and aggravated synovitis in osteoarthritis. Cell Death Dis. 10 (7), 522. 10.1038/s41419-019-1739-2 31285423 PMC6614358

[B78] Northrop-Albrecht E. J. Taylor W. R. Huang B. Q. Kisiel J. B. Lucien F. (2022). Assessment of extracellular vesicle isolation methods from human stool supernatant. J. Extracell. Vesicles 11 (4), e12208. 10.1002/jev2.12208 35383410 PMC8980777

[B79] Otto E. Knapstein P. R. Jahn D. Appelt J. Frosch K. H. Tsitsilonis S. (2020). Crosstalk of brain and bone-clinical observations and their molecular bases. Int. J. Mol. Sci. 21 (14), 4946. 10.3390/ijms21144946 32668736 PMC7404044

[B80] Park Y. S. Kim H. W. Hwang J. H. Eom J. Y. Kim D. H. Park J. (2023). Plum-derived exosome-like nanovesicles induce differentiation of osteoblasts and reduction of osteoclast activation. Nutrients 15 (9), 2107. 10.3390/nu15092107 37432256 PMC10180726

[B81] Peng J. Yu X. J. Yu L. L. Tian F. W. Zhao J. X. Zhang H. (2021). The influence of gut microbiome on bone health and related dietary strategies against bone dysfunctions. Food Res. Int. 144, 110331. 10.1016/j.foodres.2021.110331 34053534

[B82] Pepe J. Rossi M. Battafarano G. Vernocchi P. Conte F. Marzano V. (2022). Characterization of extracellular vesicles in osteoporotic patients compared to osteopenic and healthy controls. J. Bone Min. Res. 37 (11), 2186–2200. 10.1002/jbmr.4688 36053959 PMC10086946

[B83] Poos S. E. M. Mv E. Bajav O. Leeuwenburgh S. C. G. Lanao R. P. F. Hv G. (2025). Evaluation of bone adhesive barrier membranes for guided bone regeneration: an experimental study in rats. Eur. Cell Mater 51, 120–135. 10.22203/eCM.v051a07

[B84] Raggatt L. J. Partridge N. C. (2010). Cellular and molecular mechanisms of bone remodeling. J. Biol. Chem. 285 (33), 25103–25108. 10.1074/jbc.R109.041087 20501658 PMC2919071

[B85] Rashidi N. Liu C. Guillot P. V. Tamaddon M. (2025). Isolation, characterization, and *in vitro* cell studies of plant-based exosome-like nanovesicles for treatment of early osteoarthritis. Int. J. Mol. Sci. 26 (5), 2211. 10.3390/ijms26052211 40076829 PMC11900001

[B86] Reid I. R. Billington E. O. (2022). Drug therapy for osteoporosis in older adults. Lancet. 399 (10329), 1080–1092. 10.1016/s0140-6736(21)02646-5 35279261

[B87] Richardson M. K. Liu K. C. Mayfield C. K. Kistler N. M. Christ A. B. Heckmann N. D. (2023). Complications and safety of simultaneous bilateral total knee arthroplasty: a patient characteristic and comorbidity-matched analysis. J. Bone Jt. Surg. Am. 105 (14), 1072–1079. 10.2106/jbjs.23.00112 37418542

[B88] Savcı Y. Kırbaş O. K. Bozkurt B. T. Abdik E. A. Taşlı P. N. Şahin F. (2021). Grapefruit-derived extracellular vesicles as a promising cell-free therapeutic tool for wound healing. Food Funct. 12 (11), 5144–5156. 10.1039/d0fo02953j 33977960

[B89] Seo K. Yoo J. H. Kim J. Min S. J. Heo D. N. Kwon I. K. (2023). Ginseng-derived exosome-like nanovesicles extracted by sucrose gradient ultracentrifugation to inhibit osteoclast differentiation. Nanoscale 15 (12), 5798–5808. 10.1039/d2nr07018a 36857681

[B90] Serhal L. Lwin M. N. Holroyd C. Edwards C. J. (2020). Rheumatoid arthritis in the elderly: characteristics and treatment considerations. Autoimmun. Rev. 19 (6), 102528. 10.1016/j.autrev.2020.102528 32234572

[B91] Sgritta M. Dooling S. W. Buffington S. A. Momin E. N. Francis M. B. Britton R. A. (2019). Mechanisms underlying microbial-mediated changes in social behavior in mouse models of autism spectrum disorder. Neuron 101 (2), 246–259.e6. 10.1016/j.neuron.2018.11.018 30522820 PMC6645363

[B92] Siva Venkatesh I. P. Majumdar A. Basu A. (2024). Prophylactic administration of gut microbiome metabolites abrogated microglial activation and subsequent neuroinflammation in an experimental model of Japanese encephalitis. ACS Chem. Neurosci. 15 (8), 1712–1727. 10.1021/acschemneuro.4c00028 38581382

[B93] Song H. Chen X. Hao Y. Wang J. Xie Q. Wang X. (2022). Nanoengineering facilitating the target mission: targeted extracellular vesicles delivery systems design. J. Nanobiotechnology 20 (1), 431. 10.1186/s12951-022-01638-9 36175866 PMC9524104

[B94] Su X. Wang H. Li Q. Chen Z. (2025). Extracellular vesicles: a review of their therapeutic potentials, sources, biodistribution, and administration routes. Int. J. Nanomedicine 20, 3175–3199. 10.2147/ijn.S502591 40098717 PMC11913029

[B95] Thomas B. L. Montero-Melendez T. Oggero S. Kaneva M. K. Chambers D. Pinto A. L. (2024). Molecular determinants of neutrophil extracellular vesicles that drive cartilage regeneration in inflammatory arthritis. Arthritis Rheumatol. 76 (12), 1705–1718. 10.1002/art.42958 39041647 PMC11605269

[B96] Thoo L. Noti M. Krebs P. (2019). Keep calm: the intestinal barrier at the interface of peace and war. Cell Death Dis. 10 (11), 849. 10.1038/s41419-019-2086-z 31699962 PMC6838056

[B97] van Niel G. Carter D. R. F. Clayton A. Lambert D. W. Raposo G. Vader P. (2022). Challenges and directions in studying cell-cell communication by extracellular vesicles. Nat. Rev. Mol. Cell Biol. 23 (5), 369–382. 10.1038/s41580-022-00460-3 35260831

[B98] Wang T. Mo L. Ou J. Fang Q. Wu H. Wu Y. (2022). *Proteus mirabilis* vesicles induce mitochondrial apoptosis by regulating miR96-5p/Abca1 to inhibit osteoclastogenesis and bone loss. Front. Immunol. 13, 833040. 10.3389/fimmu.2022.833040 35242136 PMC8885728

[B99] Wang Z. X. Lin X. Cao J. Liu Y. W. Luo Z. W. Rao S. S. (2024). Young osteocyte-derived extracellular vesicles facilitate osteogenesis by transferring tropomyosin-1. J. Nanobiotechnology 22 (1), 208. 10.1186/s12951-024-02367-x 38664789 PMC11046877

[B100] Wang W. Liu X. Nan H. Li H. Yan L. (2025). Specific gut microbiota and serum metabolite changes in patients with osteoarthritis. Front. Cell Dev. Biol. 13, 1543510. 10.3389/fcell.2025.1543510 40027098 PMC11868077

[B101] Wang K. Li X. Huang K. Wu H. Tan M. Su W. (2025). Dietary probiotic-derived extracellular vesicles as delivery systems of bioactive compounds to maintain intestinal homeostasis. Chem. Eng. J. 505, 159546. 10.1016/j.cej.2025.159546

[B102] Welsh J. A. Goberdhan D. C. I. O'Driscoll L. Buzas E. I. Blenkiron C. Bussolati B. (2024). Minimal information for studies of extracellular vesicles (MISEV2023): from basic to advanced approaches. J. Extracell. Vesicles 13 (2), e12404. 10.1002/jev2.12404 38326288 PMC10850029

[B103] Woo H. E. Cho J. Y. Lim Y. H. (2024). Propionibacterium freudenreichii MJ2-derived extracellular vesicles inhibit RANKL-induced osteoclastogenesis and improve collagen-induced rheumatoid arthritis. Sci. Rep. 14 (1), 24973. 10.1038/s41598-024-76911-y 39443658 PMC11500175

[B104] Wu Y. Ai H. Zou Y. Yang Q. Dou C. Xu J. (2023). Osteoclast-derived extracellular miR-106a-5p promotes osteogenic differentiation and facilitates bone defect healing. Cell Signal 102, 110549. 10.1016/j.cellsig.2022.110549 36464103

[B105] Wu Q. Kan J. Fu C. Liu X. Cui Z. Wang S. (2024). Insights into the unique roles of extracellular vesicles for gut health modulation: mechanisms, challenges, and perspectives. Curr. Res. Microb. Sci. 7, 100301. 10.1016/j.crmicr.2024.100301 39525958 PMC11550031

[B106] Xu T. Xu M. Bai J. Lin J. Yu B. Liu Y. (2019). Tenocyte-derived exosomes induce the tenogenic differentiation of mesenchymal stem cells through TGF-β. Cytotechnology 71 (1), 57–65. 10.1007/s10616-018-0264-y 30599073 PMC6368508

[B107] Yadegar A. Bar-Yoseph H. Monaghan T. M. Pakpour S. Severino A. Kuijper E. J. (2024). Fecal microbiota transplantation: current challenges and future landscapes. Clin. Microbiol. Rev. 37(2):e00060-e00122. 10.1128/cmr.00060-22 38717124 PMC11325845

[B108] Yan J. Herzog J. W. Tsang K. Brennan C. A. Bower M. A. Garrett W. S. (2016). Gut microbiota induce IGF-1 and promote bone formation and growth. Proc. Natl. Acad. Sci. U. S. A. 113 (47), E7554–E7563. 10.1073/pnas.1607235113 27821775 PMC5127374

[B109] Yan L. Liu G. Wu X. (2021). Exosomes derived from umbilical cord mesenchymal stem cells in mechanical environment show improved osteochondral activity via upregulation of LncRNA H19. J. Orthop. Transl. 26, 111–120. 10.1016/j.jot.2020.03.005 33437630 PMC7773952

[B110] Yang X. Chang T. Yuan Q. Wei W. Wang P. Song X. (2022). Changes in the composition of gut and vaginal microbiota in patients with postmenopausal osteoporosis. Front. Immunol. 13, 930244. 10.3389/fimmu.2022.930244 36032115 PMC9411790

[B111] Yang K. L. Mullins B. J. Lejeune A. Ivanova E. Shin J. Bajwa S. (2024). Mitigation of osteoclast-mediated arthritic bone remodeling by short chain fatty acids. Arthritis Rheumatol. 76 (4), 647–659. 10.1002/art.42765 37994265 PMC10965381

[B112] Yin B. Ni J. Witherel C. E. Yang M. Burdick J. A. Wen C. (2022). Harnessing tissue-derived extracellular vesicles for osteoarthritis theranostics. Theranostics 12 (1), 207–231. 10.7150/thno.62708 34987642 PMC8690930

[B113] Yuan F. Peng W. Yang Y. Xu J. Liu Y. Xie Y. (2023). Endothelial progenitor cell-derived exosomes promote anti-inflammatory macrophages via SOCS3/JAK2/STAT3 axis and improve the outcome of spinal cord injury. J. Neuroinflammation 20 (1), 156. 10.1186/s12974-023-02833-7 37391774 PMC10314438

[B114] Zhan W. Deng M. Huang X. Xie D. Gao X. Chen J. (2023). Pueraria lobata-derived exosome-like nanovesicles alleviate osteoporosis by enhacning autophagy. J. Control Release 364, 644–653. 10.1016/j.jconrel.2023.11.020 37967723

[B115] Zeng H. Guo S. Ren X. Wu Z. Liu S. Yao X. (2023). Current strategies for exosome cargo loading and targeting delivery. Cells 12 (10), 1416. 10.3390/cells12101416 37408250 PMC10216928

[B116] Zeng C. Wei J. Persson M. S. M. Sarmanova A. Doherty M. Xie D. (2018). Relative efficacy and safety of topical non-steroidal anti-inflammatory drugs for osteoarthritis: a systematic review and network meta-analysis of randomised controlled trials and observational studies. Br. J. Sports Med. 52 (10), 642–650. 10.1136/bjsports-2017-098043 29436380 PMC5931249

[B117] Zeng Y. Yu S. Lu L. Zhang J. Xu C. (2024). Ginger-derived nanovesicles attenuate osteoarthritis progression by inhibiting oxidative stress via the Nrf2 pathway. Nanomedicine (Lond). 19 (28), 2357–2373. 10.1080/17435889.2024.2403324 39360651 PMC11492688

[B118] Zhan M. Zhao C. Han Y. Xiao H. (2026). From isolation to function: a review of plant-derived exosome-like nanoparticles. Trends Food Sci. and Technol. 171, 105643. 10.1016/j.tifs.2026.105643

[B119] Zhang L. Wang Q. Su H. Cheng J. (2021). Exosomes from adipose derived mesenchymal stem cells alleviate diabetic osteoporosis in rats through suppressing NLRP3 inflammasome activation in osteoclasts. J. Biosci. Bioeng. 131 (6), 671–678. 10.1016/j.jbiosc.2021.02.007 33849774

[B120] Zhang Y. W. Li Y. J. Lu P. P. Dai G. C. Chen X. X. Rui Y. F. (2021). The modulatory effect and implication of gut microbiota on osteoporosis: from the perspective of “brain-gut-bone” axis. Food Funct. 12 (13), 5703–5718. 10.1039/d0fo03468a 34048514

[B121] Zhang R. Li H. Mu Y. Li R. Li X. Gao T. (2025). Turmeric-derived extracellular vesicles loaded microneedle system attenuates rotator cuff degeneration by orchestrating energetic metabolism. Mater. Today Bio 35, 102590. 10.1016/j.mtbio.2025.102590 41431730 PMC12718207

[B122] Zhao X. Sun Z. Xu B. Duan W. Chang L. Lai K. (2023). Degenerated nucleus pulposus cells derived exosome carrying miR-27a-3p aggravates intervertebral disc degeneration by inducing M1 polarization of macrophages. J. Nanobiotechnology 21 (1), 317. 10.1186/s12951-023-02075-y 37667246 PMC10478255

[B123] Zou J. Yang W. Cui W. Li C. Ma C. Ji X. (2023). Therapeutic potential and mechanisms of mesenchymal stem cell-derived exosomes as bioactive materials in tendon-bone healing. J. Nanobiotechnology 21 (1), 14. 10.1186/s12951-023-01778-6 36642728 PMC9841717

[B124] Zu M. Xie D. Canup B. S. B. Chen N. Wang Y. Sun R. (2021). Green' nanotherapeutics from tea leaves for orally targeted prevention and alleviation of colon diseases. Biomaterials 279, 121178. 10.1016/j.biomaterials.2021.121178 34656857

[B125] Zu M. Liu G. Xu H. Zhu Z. Zhen J. Li B. (2024). Extracellular vesicles from nanomedicine-trained intestinal microbiota substitute for fecal microbiota transplant in treating ulcerative colitis. Adv. Mater 36 (41), e2409138. 10.1002/adma.202409138 39073205

